# Antibiotic Resistance in Selected Emerging Bacterial Foodborne Pathogens—An Issue of Concern?

**DOI:** 10.3390/antibiotics12050880

**Published:** 2023-05-09

**Authors:** Katarzyna Grudlewska-Buda, Justyna Bauza-Kaszewska, Natalia Wiktorczyk-Kapischke, Anna Budzyńska, Eugenia Gospodarek-Komkowska, Krzysztof Skowron

**Affiliations:** 1 Department of Microbiology, Ludwik Rydygier Collegium Medicum in Bydgoszcz, Nicolaus Copernicus University in Toruń, 85-094 Bydgoszcz, Poland; 2 Department of Microbiology and Food Technology, Bydgoszcz University of Science and Technology, 85-029 Bydgoszcz, Poland

**Keywords:** foodborne pathogens, antibiotic resistance, multidrug resistance

## Abstract

Antibiotic resistance (AR) and multidrug resistance (MDR) have been confirmed for all major foodborne pathogens: *Campylobacter* spp., *Salmonella* spp., *Escherichia coli* and *Listeria monocytogenes.* Of great concern to scientists and physicians are also reports of antibiotic-resistant emerging food pathogens—microorganisms that have not previously been linked to food contamination or were considered epidemiologically insignificant. Since the properties of foodborne pathogens are not always sufficiently recognized, the consequences of the infections are often not easily predictable, and the control of their activity is difficult. The bacteria most commonly identified as emerging foodborne pathogens include *Aliarcobacter* spp., *Aeromonas* spp., *Cronobacter* spp., *Vibrio* spp., *Clostridioides difficile*, *Escherichia coli*, *Mycobacterium paratuberculosis*, *Salmonella enterica*, *Streptocccus suis*, *Campylobacter jejuni*, *Helicobacter pylori, Listeria monocytogenes* and *Yersinia enterocolitica.* The results of our analysis confirm antibiotic resistance and multidrug resistance among the mentioned species. Among the antibiotics whose effectiveness is steadily declining due to expanding resistance among bacteria isolated from food are β-lactams, sulfonamides, tetracyclines and fluoroquinolones. Continuous and thorough monitoring of strains isolated from food is necessary to characterize the existing mechanisms of resistance. In our opinion, this review shows the scale of the problem of microbes related to health, which should not be underestimated.

## 1. Introduction

The food production environment ensures constant access to a rich source of nutrients enabling bacterial growth. The presence of microorganisms in the majority of food products is undesirable and affects food quality and consumer safety. The undoubted problem of excessive and uncontrolled microbial growth in food is its spoilage as a result of saprotroph activity. A much greater risk accompanies the emergence of pathogens responsible for food poisoning. Annually, the consumption of contaminated food products causes illness in 600 million people worldwide, and the resulting economic losses exceed USD 100 billion [1].

**Figure 1:**
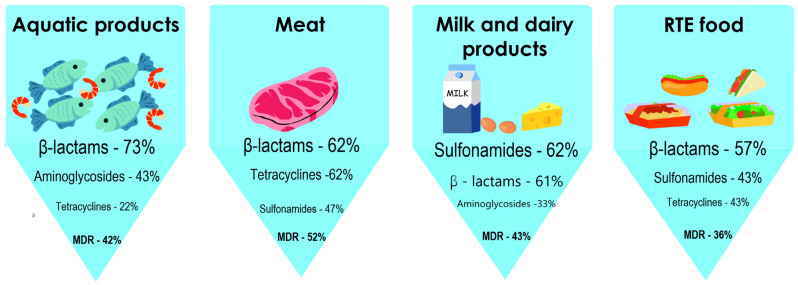
The most common types of antibiotic resistance depending on the type of food product.

The pathogenic potential is the main and obvious cause of the risks posed by the emergence of pathogens in food and its production area. Of great interest are pathogens’ properties, e.g., biofilm formation ability or persistence, which significantly complicate the control of the epidemiological situation in food production facilities [2,3,4]. Highly relevant is the phenomenon of antibiotic resistance, commonly detected in bacteria isolated from various food products [5]. An analysis of the results of studies on the antibiotic resistance of pathogens isolated from various food products indicates that the average frequency of their occurrence in food is ≥11%. Most pathogens show resistance to β-lactam antibiotics, while multidrug resistance (MDR) has been observed among ≥36% of pathogens [3] (Figure 1).

Antibiotic resistance was confirmed for all major foodborne pathogens: *Campylobacter* spp. [6,7], *Salmonella* spp. [8,9,10], *Escherichia coli* [11,12,13] and *Listeria monocytogenes* [14,15]. The prevalence of these microorganisms in food and the consequences of poisoning caused by them have resulted in a large number of studies on their activity, their preference for environmental parameter values, their most important virulence mechanisms or their sensitivity to various stressors, including disinfectants and antibiotics. Such studies facilitate the rapid and effective identification of foodborne pathogens and allow the development or modification of effective strategies for food poisoning control and prevention. Worrying are reports on emerging food pathogens—microorganisms that have not previously been linked to food contamination or were considered epidemiologically insignificant.

The present review aims to analyze the extent of antibiotic-resistant and multidrug-resistant phenomena among selected groups of emerging foodborne pathogens of increasing or persistently high epidemiological significance.

## 2. Emergence Phenomenon among Foodborne Pathogens

**Figure 2:**
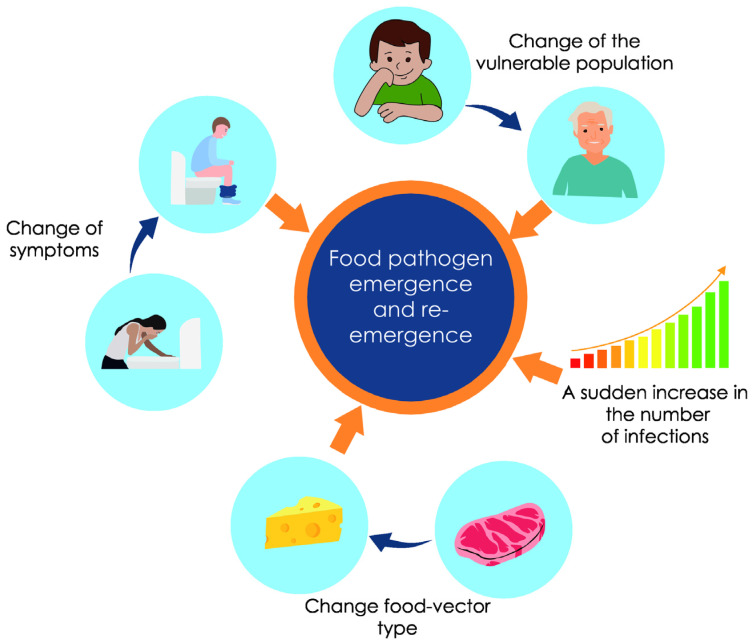
Factors indicating the presence of an emerging and re-emerging pathogens.

Defining the term emergence in relation to foodborne pathogens raised many questions, which resulted in the introduction of various subcategories or categories similar to “emerging” (Figure 2). Very often the difference between “emerging” and “new” food pathogens is emphasized, which in practice are identified rather rarely and which denote completely new, unknown species of microorganisms responsible for diseases, with a never before described course [16,17]. By “emerging” foodborne pathogens, scientists usually mean those microorganisms whose pathogenicity, although known and confirmed, has not previously been directly linked to food [17]. In contrast, “re-emerging” pathogens involve microorganisms already known as foodborne, and the risk associated with them stems from an increased frequency of poisoning cases. Rejecting the distinction between “emerging” and “re-emerging” pathogens emphasizes the possibility of emerging foodborne diseases in new host populations, in association with new types of food, or with different symptoms of infection than previously known [16,18,19]. Undoubtedly, in all these cases, they pose a threat to public health and require measures to limit their spread and minimize their impact.

Regardless of their taxonomic affiliation, the appearance of emerging pathogens in the food production environment results from the interaction of many factors, usually more or less interrelated. Some of these are directly related to informed decisions made by people, such as modifications made to farming systems, animal husbandry (e.g., animal welfare, use of antibiotics) or technological processes related to food production (e.g., introduction of new methods of food preservation) and distribution. Others, on the other hand, are socio-demographic and are associated, for example, with changes in eating habits (consumption of minimally processed or raw products), mass migration of the population (e.g., as a result of warfare), transformations in the existing structures of society (increase in the number of the so-called vulnerable consumers, i.e., people susceptible to poisoning due to age or illness) or poor quality of medical services. Moreover, evolutionary and adaptive changes contribute to acquiring previously not observed virulence traits. On the other hand, technological advances, the development and use of increasingly improved methods for identifying microorganisms, including those viable but non-culturable (VBNC), allow the identification of emerging foodborne pathogens [16,17,20,21]. All these factors induce changes that may transform microorganisms’ interaction with the human body from neutral to antagonistic.

## 3. Emerging Pathogens

The emerging foodborne pathogen category includes microorganisms and infectious agents belonging to various taxonomic units. These include parasites (*Cyclospora cayetanensis*, *Gnathostoma* spp. and *Echinococcus* spp.), viruses (hepatitis A virus, hepatitis E virus, avian influenza viruses, coronavirus and the tick-borne encephalitis virus) and bacteria. The last group appears to be critical for food microbiological safety. The bacterial genera and species most commonly identified as emerging foodborne pathogens in scientific publications include *Aliarcobacter* spp., *Aeromonas* spp., *Cronobacter* spp., *Vibrio* spp., *Clostridioides difficile*, *Escherichia coli*, *Mycobacterium paratuberculosis*, *Salmonella enterica*, *Streptocccus suis*, *Campylobacter jejuni*, *Helicobacter pylori, Listeria monocytogenes* and *Yersinia enterocolitica* [16,17,19,21,22]. While some of them, such as *Salmonella* spp. and *L. monocytogenes*, have always and unambiguously been associated with food, for others, the frequent isolation and direct association with food poisoning have been less evident. Sometimes only specific serotypes, e.g., *E. coli* STEC/EHEC and *Y. enterocolitica* serobiotype O3/4, pose increased risk [16,17,20,21,22,23]. Very important is the antibiotic sensitivity of such pathogens. Due to a lack of proven diagnostic methods and effective therapeutic strategies, the antibiotic resistance of emerging foodborne pathogens may pose a serious epidemiological threat. Foodborne illness is a serious public health threat. The most frequently reported zoonotic diseases in humans in the EU in 2021 were campylobacteriosis and salmonellosis. The report shows that the third most common disease is yersiniosis, followed by Shiga-toxin-producing Escherichia coli (STEC) and listeriosis (Table 1). In the US, the most frequent causative agent of foodborne outbreaks was *Salmonella* spp. (Table 2). The most recent outbreak of *Salmonella* infections was described by the CDC on 30 March 2023 and involved 12 cases reported in 11 different states after eating raw flour [24].

## 4. Emerging Foodborne Bacterial Pathogens—Characteristics and Antibiotic Resistance of the Most Important Species

### 4.1. Aliarcobacter spp.

The genus *Aliarcobacter* (formerly *Arcobacter*) is a Gram-negative, non-spore-forming, motile, curved bacilli of the Campylobacteraceae family, class Epsilonproteobacteria [26,27]. The genus *Aliarcobacter* currently includes nine species, *A. butzleri*, *A. lanthieri*, *A. cryaerophilus*, *A. skirrowii*, *A. thereius*, *A. cibarius*, *A. faecis*, *A. trophiarum* and *A. vitoriensis*, of which the first five are zoonotic emerging food- and waterborne pathogens [28,29]. The most widespread species with confirmed pathogenicity is *A. butzleri* [30].

The optimal growth of *Aliarcobacter* spp. occurs under microaerobic conditions and temperatures of 15–42 °C, but they also grow under aerobic and anaerobic conditions. They have been isolated from the digestive tract and feces of various livestock and wildlife, both healthy and diseased [26,31]. They are present in the environment, fresh and salt water, soil, agricultural runoff and wastewater. Researchers have detected various species of *Aliarcobacter* in food, mostly of animal origin. They were most common in poultry, pork, beef and lamb meat, milk and dairy products, fish and shellfish. Cases of *Aliarcobacter* spp. contamination of plant-based food products (fresh and ready-to-eat (RTE) vegetables) have also been reported. The ability of *Aliarcobacter* spp. to form a biofilm enables their prolonged survival and distribution in food production environments [28,32,33,34].

Consumption of products contaminated with *Aliarcobacter* spp. may lead to inflammatory bowel disease in the form of prolonged and watery diarrhea. *A. butzleri* may also cause non-diarrheal poisoning, characterized by painful abdominal cramps, fever and vomiting. In rare cases, extraintestinal illnesses such as peritonitis and bacteremia may occur [29,31,33].

The lack of adequate diagnostic recommendations indicating the need to determine the presence of these bacteria in patients with inflammatory bowel disease symptoms, inadequate identification and specific growth requirements hinder the assessment of the actual occurrence frequency of *Aliarcobacter* in the environment. The result is limited data on the properties of these microorganisms, the mechanisms of their virulence, the symptoms of the infection, the prevention and the treatment. Researchers have also investigated the antibiotic resistance of these bacteria [28,33,35].

#### Antimicrobial Resistance of *Aliarcobacter* spp.

Infections caused by pathogenic *Aliarcobacter* species do not generally require antibiotic therapy. In severe cases, fluoroquinolones and tetracyclines are the best treatment option [34,36].

The currently observed increasing frequency of antibiotic-resistant *Aliarcobacter* spp. strains, likely a result of antibiotic use in animal husbandry and medicine, may soon pose a serious epidemiological problem. Due to the lack of standardized AST (antimicrobial susceptibility testing) methods for *Aliarcobacter* spp., researchers use different techniques for its determination, which hinders obtaining data for comparative analyses [33,34,37].

Kayman et al. [38] also revealed the ampicillin resistance of *A. butzleri* strains from patients with acute gastrointestinal infection. Similar results were shown in the study by Van den Abeele et al. [39], in which only 9% of 106 strains of *A. butzleri* (63) and *A. cryaerophilus* (43) were sensitive to the antibiotic. Fanelli et al. [40] demonstrated resistance to β-lactam antibiotics (cefotaxime, penicillin, ampicillin), vancomycin, erythromycin and tetracyclin in two *A. butzleri* LMG 10828T isolates from shellfish. The genes responsible for the resistance to selected antibiotics that occurs in *Aliarcobacter* spp. are presented in Table 3. A very high frequency of erythromycin and tetracycline resistance, exceeding 95%, was observed among *A. butzleri* strains isolated from food products by Yesilmen et al. [26] and Vicente-Martins et al. [41]. On the other hand, in a study by Šilha et al. [36], 78% of *A. butzleri* from poultry meat and 100% from water were sensitive to tetracycline. Jehanne et al. [37] also found a lack of specific resistance markers for erythromycin and tetracycline in clinical strains of *A. butzleri*. Moreover, Kayman et al. [38] and Van den Abeele et al. [39] confirmed the sensitivity of this species to tetracycline in isolates from individuals with gastrointestinal disease. In our opinion, due to the conflicting results on the effectiveness of tetracycline against *Aliarcobacter* spp., the recommendation of its use as first-line treatment may raise reasonable doubts.

Scientists have revealed that the drug susceptibility of *Aliarcobacter* spp. is diverse depending on the origin of the isolates (water, food, animals, humans). At the same time, most of the results indicate a trend in the continued sensitivity of *Aliarcobacter* spp. to aminoglycosides, especially gentamicin [31,37,38,39,40,42].

A significant epidemiological problem is the emergence of multidrug-resistant (MDR) strains among *Aliarcobacter*. According to Šilha et al. [36], as many as 94.5% of *A. butzleri* isolates from poultry meat were resistant to three or more antibiotics. Vicente-Martins et al. [41] observed MDR in 85.7% of *Aliarcobacter* spp. isolates from RTE vegetables, meat and fish. However, the risk associated with the prevalence of MDR among these bacteria is sometimes questioned due to the lack of a standardized and unified method for determining their antibiotic resistance, as described above [37].

**Table 3:** Antibiotic resistance genes in selected types of bacteria.

Genus/Species	Resistance to	Genes	References
*Aliarobacter* spp.	tetracycline	*tetA, tetO, tetW*	[43]
	quinolones	*gnrS, gyrA*	[44]
	fluorochinolones (especially ciprofloxacin)	*gyrA*	[45]
	beta-lactamases	*bla1, bla2*	[42,46]
	ampicilin	*bla2*	[46]
	chloramfenicol	*cat3*	
	macrolides	*macA1, macB2*	[40]
	polymyxin	*arnB, eptA*	
	various classes of antibiotics	*rlmN*	
	suspected to be involved in multidrug resistance	*hipA*	[47]
*Aeromonas* spp.	streptomycin	*aadA1*	[48,49]
	spectinomycin	*aadA2*	
	streptothricin	*sat1*	
	tetracycline	*tetA, tetB, tettC, tetD, tetE, tetH, tetG, tetM*	[50,51,52]
	quinolone	*qnrS2, parC,* mutation *in gyrA*	
			[53]
	sulphonamide	*sul1, sul2*	[54,55]
	aminoglycosides	*aac (6′)-Ib-cr*	
	beta-lactam	*blaKPC-2, blaP1, bla_TEM_, bla_VEB-1a_, bla_SHV-12_*	
	ciprofloxacin	*aac(6′)-ib-cr*	[55]
	trimethoprim	*dfrA1, dfrA1/7, dfrA12*	[56]
	aminoglycosides	*aadA1a, aadA2, aadA7, aacA4,* *aacA, strA-strB*	[57,58,59,60]
*Escherichia coli*	β-lactams	*bla_CTX-M-1_, bla_CTX-M-14_, bla_TEM-52_, bla_SHV-12_, bla_CTX-M_, bla_TEM_, bla_SHV_* (ESBL genes)	[13,61]
	carbapenems	*bla_NDM-1_, bla_NDM-5_, bla_VIM-1_, bla_IMP-4_, bla_OXA-48_, bla_OXA-181_, bla_KPC-2_*	[62,63,64,65,66,67]
	quinolones	*gyrA*	[68]
	aminoglycosides	*armA*	[69]
	fosfomycin	mutations in the *glpT* and *uhpA/T* genes, *fosA*	[70,71]
	tetracycline	*tet(A),* *tet(B), tet(C), tet(D), tet(E), tet(G), tet(J), tet(L), tet(Y)*	[72]
	phenicols	*cmlA, floR, cfr*	[73]
	sulphonamide	*sul1, sul2, sul3*	[74]
	trimethoprim	*dfrA, dfrB*	[75]
	polymyxin	*pmrCAB*	[76]
*Salmonella* spp.	β-lactams	*bla_TEM_, bla_CTX-M_*	[77,78]
	aminoglycosides	*aac(3)-IV,* *aac(60)-Iaa, aadA1, aadA2*	[79]
	sulfonamides	*sul, dfrA1, dfrA12*	[80]
	tetracyclines	*tetA, tetB*	[81]
	quinolones	*oqxAB, qnrA, qnrB, qnrC, qnrD*, *qnrS, aac(6′)lb-cr*	[82]
	chloramphenicol	*cmlA, catB*	
	colistin	*mcr-1, mcr-3*	[83,84]
*Vibrio* spp.	aminoglycoside	*str*	[85]
	β-lactams	*blA_OXA_, bla_PSE_, ampC*	[86]
	tetracycline	*tetA, tetE*	[87,88]
	sulfonamide	*sul1, sul2*	
	quinolone	*qnr*	[89]
	chloramphenicol	*cat, floR*	[87]
	macrolides	*erm, mef, aac, aphA*	[90]
*Campylobacter* spp.	ciprofloxacin	mutation in *gyrA* gene	[91,92]
	tetracycline	*tetA, tetB, tetC, tetD, tetK, tetM*	
	erythromycin	*ermM*	
	chloramphenicol	*catI, catII*	[92]
	gentamycin	*aac(3)-IIa-(aacC2)*	
	ampicillin	*ampC*	
	imipenem	*imi, vim, kpc*	
*Cronobacter* spp.	colistin	*mcr-1, mcr-10, mcr-9.1*	[93,94,95,96]
	β-lactams	*bla_TEM_, bla_OXA_, bla_SHV_, bla_CTX-M-1_, bla_CTX-M-2_,* *bla_CTX-M-8_, bla_CTX-M-9_*	[97,98]
	fosfomycin	*glpT*	[98]
	cephalothin	*bla_CSA_*	[96]
	fluoroquinolone	*marA, marR, adeF, emrR, emrB*	
	nitroimidazole	*msbA*	
	macrolide	*kpnE, kpnF, kpnH*	
	aminoglycoside	*baeR*	
*Listeria monocytogenes*	fosfomycin	*fosX*	[99]
	lincosamides	*lin*	
	quinolones	*norB*	
	tetracyclines	*tetA, tetC, tetM, tetS*	
	vancomycin	*nacC, vanR, vanT, vanXY-C*	[100,101]
	lincomycin	*abc-f*	[101,102]
	trimethoprim	*drfE*	[101]
	macrolide, linesoide and streptogramin B	*ermB, ermC*	[101,103,104]
	β-lactams	*blaTEM-116*	[101]
	macrolide	*mphB*	
*Staphylococcus aurues*	penicillins	*blaZ*	[105,106]
	beta-lactams (MRSA)	*mecA, mecC*	[107]
	aminoglycoside	*aac(6′)/aph(2″), aph(3′)-IIIa, ant(4′)-Ia*	[108,109,110]
	macrolides, lincosamides, streptogramin B	*ermA, ermB, ermC, ermY*	[111,112]
	macrolides	*msrA, msrB, mphC*	[113]
	linezolid	*cfr* *, optrA, poxtA*	[114]
	tetracycline	*tetK, tetM*	[111]
	vancomycin	*vanA*	[115]
	fluoroquinolones	*norA*	[116]
	trimethoprim	*dfrA*	[116]
*Streptococcus suis*	tetracyclines	*tetM, tetO, tetQ, tetT, tetW, tetK, tetL*	[117]
	macrolides	*ermB, ermA, ermTR*	[118]
	lincosamides	*lnu(B), lnu(C)*	[117]
	aminoglycosides (including kanamycin and neomycin)	*aph(3′)-IIIa*	[117]
	vancomycin	*vanG*	[119,120]
	amphenicols	*cfr, cat*	[121,122]
*Clostridioides difficilie*	β-lactams	*CDD1, CCD2*	[123]
	aminoglycosides	*aph, aac, ant, npmA*	[124]
	tetracyclines	*tet44, tetM, tetW, tetA, tetB*	[125,126,127]
	vancomycin	*murG, vanS/, vanG*	[125,128,129]
	metronidazole	*glyC, nifJ*	[130,131]
	fidaxomicin	*rpoB*	[128,132,133]
	rifamycins	*rdxA*	[134,135]
	fluorochinolones	*gyrA, gyrB*	[136]
	chloramfenicol	*cat(P), cat(D)*	[125]
	linezolid	*cfrC, rplC*	[134]
*Helicobacter pylori*	metronidazole	*rdxA*	[137,138]
	amoxicillin	*pbp1A*	
	fluoroquinolone (especially levofloxacin)	*gyrA, gyrB*	
	clarithromycin	*cla*	

ESBL—extended-spectrum β-lactamases; MRSA—methicillin-resistant *Staphylococcus aureus*.

### 4.2. Aeromonas spp.

Bacteria of the genus *Aeromonas* are Gram-negative, non-spore-forming bacilli belonging to the Aeromonadaceae family and the class Gammaproteobacteria family. Facultatively anaerobic, however, they show variation in their optimal growth temperatures. The group of psychrophiles includes non-motile species, growing at 22–25 °C, while motile mesophiles have a temperature optimum of 35–37 °C. The latter group was associated with human diseases [17,139,140]. Pathogenic to humans are *A. hydrophila*, *A. caviae* and *A. veronii* [141,142].

The natural habitat of these bacteria is water sources—both salty and fresh, as well as sewage water. It explains the presence of *Aeromonas* spp. in fish and seafood, which eaten raw may pose a serious threat of food poisoning [139,141,143]. Moreover, the microorganisms were isolated from fresh vegetables, milk and poultry, pork and beef. Of concern from an epidemiological standpoint is the ability, observed in some *Aeromonas* species, to survive under refrigeration, posing a potential risk to consumers [17,144]. Pathogenic *Aeromonas* are particularly dangerous for immunocompromised people and children under two years old. Poisoning can result primarily in gastroenteritis of varying severity, but wound infections involving *Aeromonas* are also dangerous. They can cause respiratory infections, peritonitis and, in extreme cases, sepsis [17,139,141].

#### Antimicrobial Resistance of *Aeromonas* spp.

Infections caused by *Aeromonas* spp. require antibiotic therapy only in exceptional cases, as they are generally self-limiting, lasting up to one week. Nonetheless, bacteria of this genus produce β-lactamases and are naturally competent for genetic element intake through transduction and conjugation [141,145,146] (Table 3). According to Chen et al. [145], resistance to ampicillin and amoxicillin is typical of most isolates of the human pathogenic species *A. hydrophila*, *A. caviae* and *A. veronii*. In contrast, resistance to cephalothin was confirmed only in *A. hydrophila* and *A. caviae*, while *A. veronii* were sensitive to the antibiotic. A study by Ghenghesh et al. [143] demonstrated a tetracycline resistance frequency of more than 30% in *Aeromonas* spp. strains isolated from poultry meat. The tested strains, however, were sensitive to ciprofloxacin and third-generation cephalosporins. These antibiotics show high inhibitory efficacy against *Aeromonas.* Studies also focus on the possibility of using trimethoprim-sulfamethoxazole as Tekedar et al. [141] reported a low frequency of genes conferring sulfamethoxazole resistance in the *Aeromonas,* but these bacteria may have genes for resistance to this antibiotic and have been described by other authors (Table 3).

### 4.3. Escherichia coli—Various Pathotypes

Gram-negative *E. coli* rods, belonging to the Enterobacteriaceae family and the class Gammaproteobacteria, are facultative anaerobes that inhabit the digestive tract of humans and animals. The species is considered the best studied and described by scientists. However, the high evolutionary variability of the genome has resulted in strains of previously unknown characteristics. Human pathogenic *E. coli* pathotypes include two groups—extraintestinal pathogenic *E. coli* (ExPEC) and diarrheagenic *E. coli* (DEC). Within the latter group, of particular importance in the context of the emergence of food pathogens are the following pathotypes: enteropathogenic *E. coli* (EPEC), enterohemorrhagic *E. coli* (EHEC), enterotoxigenic *E. coli* (ETEC), enteroaggregative *E. coli* (EAEC), enteroinvasive *E. coli* (EIEC) and diffusely adherent *E. coli* (DAEC) [147]. In addition, some *E. coli* strains, identified as hybrid pathotypes, possess virulence genes associated with several different pathotypes, resulting in elevated virulence and difficult diagnosis [148,149,150,151].

*E. coli* is widespread in the environment, and the strains present in the human digestive tract are harmless commensals. Pathotype infections usually result from the consumption of fecally contaminated water or food. Products most frequently implicated in pathogenic *E. coli* poisoning include fruits and vegetables (sprouts, lettuce, spinach, beets, tomatoes, apple juice), poultry, pork, beef, fish and milk [16,17,147].

Depending on the pathotype, the foodborne effect of *E. coli* infection can be diarrhea and inflammation (urinary tract, lungs, skin and intra-abdominal infections), leading to death in extreme cases. Shiga toxin (Stx)-producing *E. coli* strains belonging to the EHEC pathotype, the cause of many foodborne outbreaks worldwide, may lead to non-bloody or bloody diarrhea, hemorrhagic colitis (HC) and hemolytic uremic syndrome (HUS) [147,148,150]. While the best-known EHEC serotype is O157:H7, the 2011 outbreak, resulting in over 50 deaths after eating sprouts, was caused by a hybrid *E. coli* pathotype. The strain produced Stx, had characteristics typical for EAEC and was MDR. The serotype was identified as enteroaggregative hemorrhagic *E. coli* (EAHEC) and designated O104:H4 [16,152].

#### Antimicrobial Resistance of *E. coli*

Due to the high diversity of pathogenic strains and the development of multiple AMR mechanisms, antibiotic resistance in *E. coli* is a common phenomenon. Currently, isolates producing extended-spectrum β-lactamases and carbapenemases (including KPC-2, NDM and OXA-48-like), which also often exhibit resistance to cephalosporins, quinolones and aminoglycosides, appear to be the biggest problem [153,154,155,156,157,158] (Table 3).

In a study by Dembélé et al. [154], all of DEC’s clinical *E. coli* strains showed resistance to amoxicillin and a combination of amoxicillin and clavulanic acid, and more than half of them (60%) were resistant to third- and fourth-generation cephalosporins. *E. coli* ESBL strains have been isolated from various food origins and processing environments worldwide [65,69,70]. According to Canizalez-Roman et al. [159], two-thirds of DEC strains isolated from food (mainly EPEC) showed resistance to at least one of the antibiotics routinely used to treat *E. coli* infections. Resistance to tetracycline (34%), cefotaxime (30%) and ampicillin (29%) was common [159]. Mohamed et al. [160] demonstrated resistance to tetracycline in 60% of Stx-producing strains and in 100% of strains isolated from RTE vegetables. The disturbing phenomenon of resistance to quinolones, which until recently were recommended as highly effective in treating *E. coli* infections, has been observed among strains isolated from poultry and RTE foods [160,161]. However, results from other work indicate that *E. coli* lacks or has lower resistance to ciproflaxin and nalidixic acid [154,159]. Méndez-Moreno et al. [151] revealed that fecal-derived DEC strains were sensitive to meropenem. In turn, cephalosporin sensitivity was observed among all human isolates of rare STEC serotype O80:H2 [150] and ESBL *E. coli* isolates from raw foods and RTEs [158].

Many works have confirmed the multidrug resistance of the foodborne pathotypes of *E. coli* [154,155,162] (Table 3). The frequency of MDR isolates resistant to three or more antimicrobial classes exceeded 85% [158,161] and, in some cases, affected all the isolates described [150,151].

The multidrug resistance of the foodborne pathotypes of *E. coli* has been confirmed in most works on the AMR phenomenon [154,155,162]. The frequency of MDR isolates resistant to three or more antimicrobial classes of strains is either very high, such as the 85.71% reported by Sivakumar et al. [158] or the 89.7% confirmed by Ortega-Paredes et al. [161] and in many cases affects all the isolates described [150,151].

### 4.4. Salmonella spp.

*Salmonella* spp. is one of the earliest foodborne pathogens described by man. Motile, Gram-negative bacilli are facultative anaerobes growing at a temperature of 5–47 °C, with an optimum of about 37 °C [163,164]. The genus consists of two species, i.e., *S. bongori* and *S. enterica*, which include serotypes with the greatest epidemiological significance [17,165]. *Salmonella* spp. is one of the most relevant intestinal pathogens. In EU countries, for many years, it has been the second, after *Campylobacter* spp., most common cause of zoonoses, with 60,050 confirmed cases in 2021 [23].

Although *Salmonella* infections occur primarily by the fecal–oral route, they can also result from contaminated water or food consumption. The most common source of infection is poultry meat, pork, eggs, dried food, infant formulas and fruit and vegetable products [165,166].

The serological test allows the identification of individual *Salmonella* spp. serovars, while the analysis of clinical symptoms classifies the strains as typhoid (TSS) and non-typhoid (NTS) [17,166]. The TSS *S*. *typhi* and *S. paratyphi* causing typhoid fever and paratyphoid fever, respectively, are responsible for infections only in humans. In turn, NTS *S*. Enteritidis and *S.* Typhimurium contribute to human digestive tract infections, with vomiting, diarrhea and fever, usually self-limiting but may also lead to extremely dangerous bacteremia and intravascular and focal infections. The mortality rate among people infected with iNTS (invasive NTS) is about 15%, and particularly sensitive are infants and small children, malnourished people, people with reduced immunity and the elderly [167,168].

#### Antibiotic Resistance of *Salmonella* spp.

Given the long-standing level of *Salmonella* spp. infections, the emergence of drug resistance in them was unavoidable [169]. Currently, the biggest problem seems to be the growing resistance to antibiotics of non-typhoid *S. enteritidis* and *S. typimurium*. It is a result of *Salmonella* cells acquiring mobile genetic elements, such as plasmids with IncA/C, B/O, HI1, HI2, I1, N, F and P replicons, often associated with MDR [170,171].

Standard antibiotics used in iNTS infection treatment in adults include ciprofloxacin, amoxicillin, ceftriaxone, ampicillin and trimethoprim-sulfamethoxazole [172]. After the decline in susceptibility to ampicillin and trimethoprim in the 1980s, quinolones became widely used to treat *Salmonella* spp. infections, which led to increased resistance to ciprofloxacin and the emergence of MDR strains [17,170]. The study of Nadi et al. [172] documented ampicillin resistance in 100% of *S*. *typhimurium* strains derived from the feces of sick patients. On the other hand, all *S.* Typhimurium serotypes analyzed were also sensitive to ciprofloxacin and nalidixic acid. Despite various reports on the sensitivity of non-typhoid *Salmonella* strains to ciproflaxacin, an increase in the minimum inhibitory concentration (MIC) for fluoroquinolones is observed in them, which makes this phenomenon a serious epidemiological problem [173]. Currently, plasmid-mediated quinolone resistance determinants, such as *qnrA, qnrB, qnrC, qnrD*, *qnrS, aac(6′)lb-cr* and *oqxAB*, are being increasingly reported in *Salmonella* [82] (Table 3).

Another disturbing fact reported by many researchers is the emergence of extended-spectrum β-lactamase producers (ESBLs) among *S*. Enteritidis and *S.* Typhimurium strains. Isolates producing ESBLs can serve as a source of genes associated with antibiotic resistance and pathogenesis. Their transmission leads to an increase in the number of antibiotic-resistant and virulent bacterial strains and consequently also in the number of infections that are difficult to treat [174]. According to Qiao et al. [174], all 96 ESBL-producing *Salmonella* isolates from chicken carcasses were resistant to ampicillin and about 84% to nalidixic acid. In addition, one-third of the tested strains showed resistance to 11 antibiotics. In the study by Ma et al. [175], 67 out of 110 clinical and food isolates of *S*. Enteritidis that had ACSSuT MDR (ampicillin, chloramphenicol, streptomycin, sulfamethoxazole and tetracycline) were positive for ESBL genes. The same type of multidrug resistance, referred to as pentaresistance, was also confirmed in *S.* Typhimurium definitive type 104, isolated in many countries around the world and responsible for epidemic diseases [176,177]. Many studies have demonstrated the prevalence of MDR patterns among non-typhoid *Salmonella* isolates [16,169,171], which confirms the importance of developing effective treatment regimens for *Salmonella* infections.

### 4.5. Vibrio spp.

The genus *Vibrio* includes Gram-negative, facultatively anaerobic non-spore-forming, slightly curved rod-shaped bacteria belonging to the family Vibrionaceae and the class Gammaproteobacteria. Their natural habitat is mainly warm marine waters with varying salinity, estuaries and fresh waters [178,179,180]. Climate change leading to a gradual increase in seawater temperatures is considered the main reason for the increased number of *Vibrio* infections and their classification as emerging foodborne pathogens. Human pathogenic species, in addition to *V. cholerae*, primarily include *V. vulnificus* and *V. parahaemolyticus* [181,182].

While the source of *V. cholerae* is mainly contaminated water, infections with *V. vulnificus* and *V. parahaemolyticus* mainly result from eating contaminated seafood—raw, undercooked oysters, crabs and shrimp and fish (such as in sushi). Both species are considered the leading foodborne pathogens globally [178,182,183].

The symptoms of infections caused by *V. parahaemolyticus* and *V. cholerae* are similar and include acute gastroenteritis with abdominal cramps, vomiting and diarrhea. *V. vulnificus*, in addition to gastrointestinal symptoms, can cause skin and soft tissue infections. In extreme and rare cases, it can lead to sepsis and death [178,180,182].

#### Antimicrobial Resistance of *Vibrio* spp.

Intestinal infections caused by *Vibrio* spp. have a mostly benign course that does not require antibiotics, and the level of susceptibility of pathogenic strains is relatively satisfactory. Nevertheless, the rise in the number of reports on the prevalence of antibiotic resistance among foodborne *Vibrio* spp. isolates documents the growing scale of the problem. The results of many studies demonstrate the emergence of their resistance to tetracycline, aminoglycosides, cephalosporins or fluoroquinolones, which have been effective previously [178,184,185] (Table 3). According to Elmahdi et al. [183], among isolates of *V. parahaemolyticus* and *V. vulnificus* from oysters, the most frequent were resistance to cephalothin (67%) and tetracycline (29%). The same author also reported, independent of the geographic zone, the widespread occurrence in *V. parahaemolyticus* and *V. vulnificus* resistance to ampicillins, penicillins and tetracyclines [185]. Moreover, Tan et al. [186] found that *V. parahaemolyticus* isolates from seafood were sensitive to most antibiotics tested, in addition to ampicillin, penicillin and cefazolin. Over 90% of strains were resistant to more than one drug [186]. Ampicillin resistance was also a leading trend among seafood *V. parahaemolyticus* isolates studied by Yang et al. [184]. The authors noticed that 68.38% of the isolates exhibited MDR. Elmahdi et al. [183], on the other hand, observed resistance to all 20 antibiotics used in the study in 10% of isolates of *V. parahaemolyticus* and *V. vulnificus.*

### 4.6. Campylobacter spp.

*Campylobacter* spp. are motile, non-spore-forming, microaerophilic, thermophilic bacteria growing in the temperature range of 31–44 °C (optimum 42 °C). The slightly bent, cylindrical cells are characterized by shape variation and the ability to assume a spherical form [187,188,189,190]. For over a decade, campylobacteriosis has been the most frequent zoonosis in the EU. The continuing upward trend in the number of infections, likely frequently underreported, as well as the biofilm-formation ability, makes *Campylobacter* spp. emerging foodborne pathogens [188,189].

The natural reservoir of pathogenic *Campylobacter* spp. is warm-blooded animals, but they can also survive outside their bodies, such as in contaminated water. The pathogenic species for humans are primarily *C. jejuni* and *C. coli*. The main foods related to gastrointestinal infections in people are raw milk, poultry and seafood, fresh fruits and vegetables (*C. jejuni*, *C. coli* and *C. fetus*) [191].

The infectious dose sufficient to cause campylobacteriosis is only 500 cells. The infection results in acute gastrointestinal symptoms, including loose and watery or profusely bloody diarrhea, fever and abdominal cramps. Among extraintestinal manifestations, *Campylobacter* spp. can cause cardiovascular complications, arthritis and meningitis, Guillain–Barre syndrome, Miller Fisher syndrome and septicemia and bacteremia [190,192].

#### Antimicrobial Resistance of *Campylobacter* spp.

Antibiotic therapy in the treatment of campylobacteriosis is justified only in situations of extremely severe infections.

Jafari et al. [193] reported an increased prevalence of resistant *Campylobacter* spp. isolates to nalidixic acid, ciprofloxacin and tetracycline. According to El Baaboua et al. [189], 100% strains of *C. jejuni* taken from meat and its production and distribution environment were insensitive to cephalothin and were highly resistant to quinolones, including nalidixic acid (50–100%). Moreover, Lazou and Serafeim [194] confirmed resistance to this antibiotic among most meat-derived *C. jejuni* isolates (70.6–76.5%). On the other hand, *C. coli* isolates from the same source showed a much lower frequency of resistance to this drug (5.0–15.7%).

*Campylobacter* spp. are considered highly susceptible to macrolides (erythromycin and newer-generation preparations) and aminoglycosides (gentamicin and streptomycin). Nonetheless, reports on the emergence of resistance to these antibiotics are becoming more frequent. It includes the MDR phenomenon, which is increasingly common among pathogenic *Campylobacter* spp. [189,190,194,195] (Table 3).

### 4.7. Cronobacter spp.

*Cronobacter* spp. are Gram-negative, motile, non-spore-forming, facultatively anaerobic bacilli of the family Enterobacteriaceae. Species belonging to this genus were isolated from the environment, including water and soil, and various foods, such as grains, vegetables, spices, cheese, fish and meat. Of the greatest concern is powdered infant formula contaminated with *C. sakazakii*, an opportunistic pathogen responsible for life-threatening meningitis, sepsis and necrotizing enterocolitis, especially in premature and low-birth-weight infants. Recently, the emergence of infections caused by *Cronobacter* spp. in adults, especially the immunocompromised and elderly, has also been observed [16,17,196,197].

The spectrum of antibiotics effective in treating infections caused by *Cronobacter* spp. is still quite broad. However, reports on baby food strains resistant to, e.g., chloramphenicol, ampicillin, amoxicillin, tetracycline and cephalosporins, are more frequent. The spread of antibiotic resistance among foodborne isolates poses an important and controllable public health challenge [17,97,197].

### 4.8. Helicobacter pylori

*Helicobacter* genus are Gram-negative, microaerophilic bacteria belonging to the phylum Campylobacterota, family Helicobacteraceae. *H. pylori* is the best-known species, which asymptomatically colonizes the gastric mucosa of people around the world. It can also be responsible for gastritis, peptic ulcer disease and gastric cancer. It is assumed that both *H. pylori* and non-pylori species of *Helicobacter* spp. (e.g., *H. pullorum, H. canadensis*) may be considered as new foodborne pathogens. Their presence was detected in water, vegetables and animal products (raw milk, poultry, fish). Poisoning caused by their consumption was to result in diarrhea, gastroenteritis and liver diseases in humans [16,17,198].

#### Antibiotic Resistance of *H. pylori*

*H. pylori* resistance to antibiotics is a problem, with increasing concern among epidemiologists. The intensity of this phenomenon is strongly influenced by the patient’s age and sex and also shows regional differences [199]. The level of resistance of these bacteria to metronidazole and clarithromycin seems to be particularly alarming [200,201]

Among *H. pylori* strains isolated from 87 patients from three ethnic groups (Han/Tibetan/Yi), none showed resistance to amoxicillin, tetracycline and furazolidone. Overall resistance rates for metronidazole, rifampicin, clarithromycin and levofloxacin were 71.3%, 60.9%, 55.2% and 18.4%, respectively [202]. A similar trend in *H. pylori* resistance was reported by Shu et al. [203], where resistance rates to metronidazole and levofloxacin were 81.7% and 22.8%, and 9.4% of strains were susceptible to all tested antibiotics. In turn, in the research of Zhang et al. [204], the highest percentage of *H. pylori*-resistant strains obtained from volunteers was observed for levofloxacin at 44.9%, while for clarithromycin, metronidazole, amoxicillin and tetracyclines, it was 41.0%, 38.8%, 6.3% and 1.1%, respectively. According to Alba et al. [200], the phenomenon of resistance to tetracyclines and amoxicillin (encoded by gene *pbp1A*) is relatively rare. This is also confirmed by the results of Nestegard et al. [201] who did not find resistance to amoxicillin among *H. pylori* isolates from patients with confirmed presence of these microorganisms. Zerbetto De Palma et al. [137] showed that single-point mutations in the quinolone-resistance-determining region (QRDR) of *gyrA* appear to be the main event leading to fluoroquinolone resistance (Table 3).

### 4.9. Listeria monocytogenes

*Listeria monocytogenes* are Gram-positive rods belonging to the genus *Listeria*, family Listeriaceae and Class Bacilli [205]. *L. monocytogenes* are non-spore-forming and relatively anaerobic [206]. *L. monocytogenes* is tolerant to changing environmental conditions and can grow in a wide range of temperatures (0–45 °C), pH (4.3–9.6) and salinity (10.0% NaCl) [207]. Due to high adaptability, *L. monocytogenes* is widespread in the environment, including water, soil and wastewater. The major source of these pathogenic rods is food [208,209,210], and unpasteurized milk, raw fruits and vegetables, raw and smoked fish, raw meat and ready-to-eat food (RTE) are most commonly implicated [211]. In 2020, fish and fish products had the highest percentage of *L. monocytogenes* contamination in European Union countries [212]. In recent years, there have been several major listeriosis outbreaks, including those associated with ready-to-eat processed meat products in 2017–2018 in the Republic of South Africa (200 deaths) [213], in 2018 in Australia (rockmelons, 7 deaths) [214] and in 2011 in the United States (cantaloupe, 33 people deaths) [215]. A major problem associated with the contamination of food products with *L. monocytogenes* rods is their presence in the food production environment, including areas that are difficult to access for cleaning and disinfection [210,216,217]. Moreover, the ability to form a biofilm on biotic and abiotic surfaces [218,219] and the isolation of persistent strains [4,220,221] contribute to food contamination with *L. monocytogenes*.

*L. moncoytogenes* is the etiological agent of listeriosis, which has a high mortality rate of up to 30%. The most sensitive are the elderly, pregnant women, newborns and immunosuppressed individuals. Listeriosis can take two forms—non-invasive and invasive [222]. The *L. monocytogenes* infection cycle is mainly associated with genes organized on *Listeria* Pathogenicity Island 1 (LIPI-1) and the InlA-InlB locus [223]. In contrast, genes located on LIPI-4 are involved in neuroinfection and fetal infection [224].

#### Antimicrobial Resistance of *Listeria monocytogenes*

The baseline treatment option for listeriosis includes antibiotic therapy with gentamicin or ampicillin [225]. Among the recommended antibiotics for listeriosis therapy are also rifampicin, vancomycin, linezolid and carbapenem [225]. In the case of allergy to beta-lactam antibiotics, trimethoprim is used [225]. In recent years, researchers have observed resistance to various antibiotics, including those used to treat listeriosis, among strains of *L. monocytogenes*. The first strain of *L. monocytogenes* identified as multidrug-resistant was isolated from a patient with meningitis (France, 1988) [226]. Poyart-Salmeron [226] demonstrated resistance to chloramphenicol, erythromycin, streptomycin and tetracycline. Morvan et al. [227] showed antibiotic resistance among 1.27% of the tested strains of clinical origin. In turn, Fallah et al. [228] found that 11 (4.0%) isolates from fish were insensitive to one or more antibiotics. Moreover, Korsak et al. [229] noted antibiotic resistance among *L. monocytogenes* strains isolated from a Polish fish-processing plant. These strains showed resistance to rifampicin, vancomycin, ampicillin, gentamycin, erythromycin, chloramphenicol, sulfamethoxazole, amoxicillin and trimethoprim [229]. Abdollahzadeh et al. [230] noticed that all tested strains isolated from seafood and humans in Iran were resistant to ampicillin and cefotaxime, while 57.0% of the strains were insensitive to penicillin [230]. Moreover, Zhang et al. [231] showed that 73% of 167 *L. monocytogenes* isolated from retail foods were resistant to sulfonamides, and as many as 8.4% showed resistance to tetracycline and 1.8% to ciprofloxacin. Sakaridis et al. [232] showed tolerance of all (n = 55) *L. monocytogenes* strains isolated from chicken slaughterhouses to nalidixic acid and oxolinic acid, while 83.6% were resistant to clindamycin. In contrast, Wu et al. [233] found that among 248 strains of *L. monocytogenes* isolated from raw retail foods, only 59 (23.8%) were sensitive to all 14 antibiotics tested. The strains were resistant to clindamycin (46.8%) and tetracycline (10.1%) [233]. Lee et al. [234] demonstrated resistance to benzyl penicillin, clindamycin and oxacillin among all tested strains of *L. monocytogenes* isolated from RTE seafood products and food-processing environments. The acquisition of mobile genetic elements (mobilizable plasmids, self-transferable plasmids, conjugative transposon) is considered the main mechanism of antibiotic resistance development in *L. monocytogenes* [14] (Table 3).

The increase in antibiotic resistance in *L. monocytogenes* observed in recent years is a serious public health concern. It is essential to monitor the prevalence of *L. monocytogenes* in food and food-processing environments and to assess their antibiotic sensitivity. Such management can help reduce outbreaks of listeriosis associated with contaminated food.

### 4.10. Staphylococcus aureus

Belonging to the phylum Bacillota and family Staphylococcus, the species *Staphylococcus aureus* is a coagulase-positive, Gram-positive cocci, capable of forming irregular patterns, non-motile and non-endospore. The optimal temperature for their growth is 30–37 °C, and the pH is 7–7.5. They also grow at NaCl concentrations of up to 15%. *S. aureus* is considered to be one of the most important etiological agents of local and systemic nosocomial infections [235,236].

*S. aureus* is a commensal colonizing the surface of the skin and mucous membranes, mainly the nose and throat of humans and animals, but it can also survive in the natural environment—in manure, water and air [237,238]. Among the food products most often associated with the presence of these bacteria and their toxins are meat, cheese and dairy products, fish and bakery products. The cause of food poisoning caused by *S. aureus* are enterotoxins produced by them, characterized by resistance to stress factors, including high temperature [239,240].

Food poisoning caused by *S. aureus* is characterized by a short incubation period (from 30 min to 8 h), rapid course and frequent remission after 24 h. The main symptoms include abdominal pain, nausea, vomiting and diarrhea. Hospitalization, if necessary, is limited to regulating water management and the level of electrolytes in the body [241].

#### Antimicrobial Resistance of *Staphylococcus aureus*

The most epidemiologically significant phenomenon associated with antibiotic resistance of *S. aureus* is the emergence of methicillin-resistant *S. aureus* (MRSA) strains in the environment, characterized by resistance to almost all beta-lactam antibiotics, and sometimes also to many other groups of antibiotics and chemotherapeutics (Table 3). Due to the high survival rate of *S. aureus* outside the living organism (even several months), multidrug-resistant strains are a serious problem not only in the hospital environment but also in the food production environment [242].

According to the results of a global meta-analysis by Ou et al. [243], the prevalence of *S. aureus* and MRSA strains in raw meat products was 29.2% and 3.2%, respectively. According to Wang et al. [244], among 50 strains of *S. aureus* isolated from raw milk samples, 6 (12.0%) were methicillin-resistant, while the highest percentage of strains showed resistance to ampicillin and erythromycin (56.0 and 54.0%, respectively). Lv et al. [245] also confirmed the presence of methicillin-resistant gene *mec* in 9 (6.1%) out of 138 *S. aureus* strains from food, almost all of which (95.1%) had the *blaZ* gene encoding penicillinase. In the study of Seow et al. [246], MRSA isolated from food accounted for 8.0% of all 100 strains, of which 58.0% were resistant to penicillin. Only one of the 160 samples taken by Komodromos et al. [247] in meat processing plants contained the MRSA isolate, while over 68% of *S. aureus* strains were resistant to penicillins. Among the strains of *S. aureus* derived from milk and fresh soft cheese, Szczuka et al. [248] showed 28.0% of MDR strains, of which two (5.1%) were identified as MRSA. Multidrug resistance among foodborne isolates of *S. aureus* is a phenomenon repeatedly confirmed, and the frequency of their occurrence may vary from a few to several dozen percent [244,246,247,248]. A high (75.2%) percentage of MDR *S. aureus* strains was isolated by Mahros et al. [249] from 225 beef burger and hot dog sandwiches. Of particular concern, however, was the presence of four (2.1%) vancomycin-resistant *S. aureus* (VRSA) strains in the tested samples, due to the use of this antibiotic as one of the first-line drugs for the treatment of infections of MRSA etiology. Further cases of VRSA strains in RTE meat (shawarma and burger sandwiches) were shown by Saber et al. [250], who reported them in 26.7% of the tested products. In turn, Shahid et al. [251], evaluating the presence of *S. aureus* in serving utensils in food-processing environments in Mymensingh city, Bangladesh, found vancomycin resistance in 29.0% of the strains. An important problem in the treatment of infections caused by multidrug-resistant MRSA is strains resistant to linezolid (LRSA), a drug that is one of the last-resort antibiotics. Their incidence is currently relatively low, but Lienen et al. [113] isolated two methicillin-resistant LRSA strains from pig meat.

The antibiotic resistance of *S. aureus*, potential etiological agents of food poisoning, combined with the high stability of the toxins produced by them and the probable underestimation of the actual incidence of diseases caused by these bacteria (including MRSA), determines the scale of risk resulting from their presence in the food production environment and the need to control this phenomenon.

### 4.11. Streptococcus suis

*Streptococcus suis* is a Gram-positive, facultative anaerobe (type Bacillota, family Streptococcaceae) that occurs both as a commensal and as a pathogen in pigs. Infections in humans are generally identified among people associated with pig farming and meat processing. In addition to the main route of infection, which is contact with animals and being in their breeding environment, the disease can also occur after eating undercooked pork. Analyses of literature sources indicate that infections caused by *S. suis* in humans mainly affect Asian countries, but it seems that their frequency in other parts of the world, especially in Europe and the United States, may be underestimated. Human infections are mainly caused by *S. suis* serotype 2, one of 35 identified within this species, and their most common clinical manifestations are meningitis, sepsis and septic shock, often accompanied by skin lesions and hearing loss [16,252,253,254,255].

#### Antibiotic Resistance *Streptococcus suis*

The antibiotic resistance of *S. suis*, due to the extremely close relationship of these bacteria with pigs, shows variability depending on the farming system used in a given country and legal regulations regarding the possibility of using antibiotics in it. According to Uruén et al. [256], the highest values of resistance indices of these bacteria are recorded against lincosamides, macrolides and tetracyclines, while they remain relatively sensitive to β-lactams and quinolones. This is confirmed by the results of research conducted in Thailand by Yongkiettrakul et al. [257] in which swine and human strains of *S. suis* showed low susceptibility to tetracycline (5.0%), clindamycin (6.5%) and doxycycline (9.2%). Cefotaxime, ceftiofur, vancomycin and florfenicol were considered effective in the treatment of *S. suis* infections, to which 93.1%, 94.7%, 96.6% and 92.4% of the tested strains were susceptible, respectively. Resistance to tetracycline ranging from 44.35% to 66.0% was observed among *S. suis* isolates from Czech pig farms in 2018–2022 by Nedbalcova et al. [258]. Responsible for tetracycline resistance in *S. suis* are tetM, tetO, tetQ, tetT, tetW, tetK and tetL genes [117] (Table 3). Dong et al. [259] found the highest resistance of *S. suis* isolated from pigs to macrolides and the lowest to fluoroquinolones.

### 4.12. Clostridioides difficile

*Clostridioides difficile* are Gram-positive bacteria belonging to the Clostridiaceae family and phylum Bacillota (Firmicutes). They are spore-forming anaerobes that are highly sensitive to oxygen. The bacteria multiply between 25 °C and 45 °C, with the optimum at 35–37 °C [260,261,262].

*C. difficile* are widespread in the environment (soil, water) and inhabit the gastrointestinal tract of humans and animals [16,17,260,263]. The possibility of an oral transmission route of *C. difficile* has been questioned for a long time. Nonetheless, a growing number of studies definitively support this hypothesis. *C. difficile* was isolated from raw ground beef pork, poultry meat, ready-to-eat animal products (cured meats), shellfish and fish (often consumed raw) but also minimally processed fruits and vegetables [260,264].

Ingestion of food-contaminating *C. difficile* spores may lead to their germination in the small intestine and the production of toxins toxin A (TcdA) and toxin B (TcdB), causing symptoms ranging from varying degrees of diarrhea to potentially fatal colitis. Among the factors determining susceptibility to *C. difficile* infection (CDI) are, first and foremost, antibiotic exposure, old age and hospitalization or residence in nursing units [16,263,265].

#### Antimicrobial Resistance of *C. difficile*

Due to the high epidemiological significance of *C. difficile*, the antibiotic resistance of these bacteria is intensively and permanently monitored. The results of studies conducted in many regions of the world, as well as conclusions from meta-analyses of these data, unequivocally indicate an increased risk of the emergence of antibiotic resistance, especially among new isolates, generally also characterized by strong virulence [266,267]. Of concern is the emergence of resistance to quinolones among them. According to Peng et al. [268], the percentage of clinical *C. difficile* isolates resistant to gatifloxacin and moxifloxacin was 28.06 and 28.78%, respectively. Most isolates, 82.70% and 75.54%, respectively, showed resistance to clindamycin and cefoxitin, while all were sensitive to metronidazole. Moreover, in the study by Heidari et al. [269], metronidazole effectively inhibited the growth of all *C. difficile* isolates from patients with post-antibiotic diarrhea. The least effective was tetracycline, as 66.7% of strains showed resistance to this drug. Sholeh et al. [270] confirmed tetracycline resistance in one-fifth of human *C. difficile* isolates.

One of the risks associated with the use of antibiotics in the treatment of CDI is the gradual acquisition of resistance to the standard active substances used to date. Studies on *C. difficile* antibiotic susceptibility have indicated that resistance to metronidazole and vancomycin is infrequent. Lynch et al. [131] described a metronidazole-resistant *C. difficile* clinical isolate. They detected genes associated with electron transport, such as glycerol-3-phosphate dehydrogenase (*glyC*) and pyruvate-flavodoxin oxidoreductase (*nifJ*) [131] (Table 3). Disruption of electron transport alters both the energy production and intracellular redox potential which influence the efficiency of metronidazole entry and activation [59]. However, even single cases of tolerance should spur efforts to prevent or reduce this phenomenon [268,269,270]. The results of Peng et al. [268] showed that 59.71% of *C. difficile* strains were resistant to three types of antibiotics, mainly to ampicillin + cefoxitin + clindamycin (49.64%), while 4.32% were resistant to six types of antibiotics.

## 5. Conclusions

AMR remains a global burden, with significant and growing morbidity and mortality worldwide. Regional differences in the stewardship, policy and regulation of antibiotics, national surveillance and methodologies for detection, further compound the problem. The emergence of foodborne pathogens appears to be a process significantly driven by human activity and the effects of human decisions, more or less rational. Most of the emerging pathogens observed in the food chain in recent years are zoonotic. The mismanagement of antibiotics used in animal husbandry, i.e., incorrect selection or overuse, is considered one of the main causes of the emergence and transmission of antibiotic-resistant foodborne pathogens. Among the antibiotics whose effectiveness is steadily declining due to expanding resistance among bacteria isolated from food are β-lactams, sulfonamides, tetracyclines and fluoroquinolones. In addition, reports on multidrug resistance among foodborne bacteria are more frequent. Of particular concern are emerging foodborne pathogens, whose properties, specific to individual species, pathovars or strains, are not always sufficiently recognized, and the consequences of the infections they cause are often difficult to predict. Therefore, understanding the dynamics of AMR is crucial to develop improved methodologies for surveillance, gaining insight into the routes and mechanisms of transmission. Current surveillance strategies vary between countries, with researchers being most focused on diseased people. The epidemiological risks posed by pathogen emergence, effective prevention, control and proper treatment are important challenges of modern medicine. In our opinion, for this reason, it is necessary to extend the monitoring of the occurrence of microorganisms in animal production, with particular emphasis on antibiotic-resistant bacteria, including those from the ESCAPE group, but also others to prevent the spread of antibiotic resistance. Despite the introduced standards, regulations and guidelines, e.g., the WHO, it is necessary to further limit using antibiotics in healthy animals to prevent the spread of antibiotic resistance. Early and continuous access to data on antibiotic use and antimicrobial resistance will enable focused efforts to limit the build-up of AMR. Long-term control and elimination of infectious diseases in animals will contribute to reducing the use of antibiotics. In our opinion, it is also extremely important to educate animal breeders, veterinarians and food producers in this area so as to reduce the consumption of antibiotics and use them only in justified cases. We believe that modern molecular methods, such as sequencing, will make it possible to understand the transmission of antibiotic resistance genes between different species of microorganisms. In addition, the methods are fast and allow the detection of genes in microorganisms that are not phenotypically resistant but can transmit genes to other strains. Moreover, frequent testing of the resistome in the environment of crop production, animal production and food-processing plants should be sought.

## Figures and Tables

**Table 1:** Foodborne outbreaks caused by emerging and re-emerging food pathogens in EU in 2021 [23].

Causative Agent		Foodborne Outbreaks	Cases of Illness	Hospitalization	Deaths	Food Vehicles Causing Strong-Evidence Outbreaks
*Salmonella* spp.		773 (143) *	6755	1123	1	eggs and egg products (39) **, mixed food (24), bakery products (15), pig meat and products thereof (14), vegetables and juices and other products thereof (11)
*Campylobacter* spp.		249 (20)	1051	134	6	broiler meat and products thereof (7), mixed food (5), bovine meat and products thereof (3), other mixed or unspecified poultry meat and products thereof (2)
*Escherichia coli*	STEC	31 (5)	275	47	0	bovine meat and products thereof (2), milk (1), vegetables and juices and other products thereof (1), meat and meat products unspecified (1)
	other than STEC	27 (4)	327	44	0	vegetables and juices and other products thereof
*Listeria monocytogenes*		23 (8)	104	48	12	fish and fish products (4), meat and meat products unspecified (2), other or mixed red meat and products thereof (1), broiler meat and products thereof (1)
*Vibrio cholera* (non-toxigenic)		1 (1)	47	1	0	mixed food, crustaceans, shellfish, mollusks and products thereof
*Vibrio parahaemolyticus*		3 (1)	10	0	0	
*Aeromonas* spp.		1 (1)	19	0	0	mixed foods
*Cronobacter sakazakii*		1 (1)	4	4	1	hospital-mixed probiotic formula for infants
*Staphylococcus aureus* toxins		61 (20)	640	51	0	mixed foods, other, mixed and/or unspecified poultry or red meat and products thereof, fish and fish products, dairy products, vegetables and juices and other products thereof

* strong-evidence outbreaks. ** number of outbreaks related to the individual food type.

**Table 2:** Foodborne outbreaks caused by emerging and re-emerging food pathogens in USA in 2021 [24,25].

Causative Agent	Cases of Illness	Hospitalization	Deaths	Food Vehicles
*Salmonella* Thompson	115	20	0	seafood
*Salmonella* Oranienburg	1040	260	0	whole, fresh onions
*Salmonella* Typhimurium	31	4	0	packaged salad greens
*Salmonella*	9	3	0	frozen cooked shrimp
Weltevreden				
*Salmonella*	20	5	0	cashew brie
Duisburg				
*E. coli* O157:H7	47	19	1	spinach,
				packaged salad,
				unknown
*E. coli* O121	16	7	0	cake mix
*Listeria monocytogenes*	28	26	4	packaged salad

## Data Availability

No new data were created or analyzed in this study. Data sharing is not applicable to this article.
